# Straightforward Inference of Ancestry and Admixture Proportions through Ancestry-Informative Insertion Deletion Multiplexing

**DOI:** 10.1371/journal.pone.0029684

**Published:** 2012-01-17

**Authors:** Rui Pereira, Christopher Phillips, Nádia Pinto, Carla Santos, Sidney Emanuel Batista dos Santos, António Amorim, Ángel Carracedo, Leonor Gusmão

**Affiliations:** 1 IPATIMUP – Institute of Molecular Pathology and Immunology of the University of Porto, Porto, Portugal; 2 Institute of Forensic Sciences Luis Concheiro, University of Santiago de Compostela, Santiago de Compostela, Spain; 3 Faculty of Sciences, University of Porto, Porto, Portugal; 4 Mathematics Research Centre, University of Porto, Porto, Portugal; 5 Laboratório de Genética Humana e Médica, Universidade Federal do Pará, Belém, Brazil; 6 Genomics Medicine Group, CIBERER, University of Santiago de Compostela, Santiago de Compostela, Spain; Erasmus University Medical Center, The Netherlands

## Abstract

Ancestry-informative markers (AIMs) show high allele frequency divergence between different ancestral or geographically distant populations. These genetic markers are especially useful in inferring the likely ancestral origin of an individual or estimating the apportionment of ancestry components in admixed individuals or populations. The study of AIMs is of great interest in clinical genetics research, particularly to detect and correct for population substructure effects in case-control association studies, but also in population and forensic genetics studies.

This work presents a set of 46 ancestry-informative insertion deletion polymorphisms selected to efficiently measure population admixture proportions of four different origins (African, European, East Asian and Native American). All markers are analyzed in short fragments (under 230 basepairs) through a single PCR followed by capillary electrophoresis (CE) allowing a very simple one tube PCR-to-CE approach.

HGDP-CEPH diversity panel samples from the four groups, together with Oceanians, were genotyped to evaluate the efficiency of the assay in clustering populations from different continental origins and to establish reference databases. In addition, other populations from diverse geographic origins were tested using the HGDP-CEPH samples as reference data. The results revealed that the AIM-INDEL set developed is highly efficient at inferring the ancestry of individuals and provides good estimates of ancestry proportions at the population level.

In conclusion, we have optimized the multiplexed genotyping of 46 AIM-INDELs in a simple and informative assay, enabling a more straightforward alternative to the commonly available AIM-SNP typing methods dependent on complex, multi-step protocols or implementation of large-scale genotyping technologies.

## Introduction

Initial studies of human genetic variation focused on Short Tandem Repeats (STRs) and Single Nucleotide Polymorphisms (SNPs) [1], [2], and only later explored Copy Number Variants (CNVs) [3]–[6] and Insertion Deletion Polymorphisms (INDELs) [7]–[9] unveiling previously unknown sources of genetic diversity that are likely to be important factors underlying inherited traits and diseases in humans. Moreover, advances in genotyping technologies have allowed progressively higher genome coverage using resources within the normal scope of most genetics laboratories. These developments have led to an increase in Genome Wide Association Studies (GWAS) in the search for genetic variants associated with a wide range of complex diseases and phenotypic traits including, for example, obesity, schizophrenia, autism, diabetes, height, eye and skin color [10]–[12].

These investigations have identified a large number of candidate gene variants showing strong association with specific conditions or phenotypes and subsequent replication studies and meta-analysis have strengthened or weakened these initial findings. One of the major problems in case-control association studies is the presence of undetected population structure that can lead to finding false positive associations when an excess of ancestry differentiated markers stratifies the case and the control groups. Alternatively false negative results may occur if real associations are missed if weak while greater allele frequency differentiation exists between study and control groups due to differences in ancestry [13], [14]. Therefore, association studies must be accompanied by an evaluation and correction of the possible effects of population structure between both sample groups. In recent years the prevailing strategies to overcome the dangers of population stratification use genomic control to measure the possible effects of stratification and correct for such effects using methods that infer genetic ancestry, each with particular pros and cons [14]–[16]. Structured association approaches involve inferring genetic ancestry of individuals in subpopulation clusters using programs like STRUCTURE [17] and association tests are then assessed correcting for individual admixture [18]. Principal component analysis (PCA) can also be applied in genetic data to infer population structure using the top components as covariates to correct for stratification in GWAS [19]. Another strategy that has been considered is genetic matching, in which cases and controls are matched for genetic ancestry, as assessed by one of the strategies described above [14], [20]. In GWAS, using data from a large number of random genetic markers is by itself sufficient and preferable to achieve good ancestry estimates to use in subsequent correction. Nevertheless, when genome wide data are not available and only few loci are studied, such as broad-scale follow-up studies focused on regions showing associations (Phase II), a proper correction for stratification can be achieved using compact panels of ancestry-sensitive or ancestry-informative markers (AIMs) [14], [21].

AIMs show high allele frequency divergence between different ancestral or geographically distant populations and are especially useful in inferring the likely ancestral origin of an individual or estimating the apportionment of ancestry components in admixed individuals or populations. Ancestry information can then be used to perform genetic matching or correct substructure effects in case-control association studies. In the population genetics field AIMs are used mainly to estimate ancestry proportions in admixed populations and assess the structure of those populations. Furthermore, AIMs are of great interest in forensic genetics, with the potential to provide an intelligence tool in criminal investigations. In the absence of any other investigative leads, AIM genotypes obtained from evidential material could indicate the likely ancestry of the donor, and therefore help direct the course of investigations [22]–[25].

In recent years several studies have been published reporting AIM sets varying greatly in the type of polymorphism, the number of loci involved and the genotyping strategies, ranging from simple PCR followed by capillary electrophoresis (e.g. INDEL sets) to more laborious and resource-intensive technologies (e.g. SNP typing by SNaPshot and TaqMan assays). The reported AIM sets have also focused attention on different population group comparisons, depending on the ancestral contributors to the admixed populations under study, or otherwise comprise more generic panels aimed at efficient population differentiation at the continental level. The great majority of AIM panels described to date use SNPs and only a minority apply STRs [26] or INDELs [27].

In this study we followed an approach that brings together highly informative short binary INDELs that combine the desirable characteristics of the other genetic markers most commonly used [7]–[9], [27]–[29]. INDELs are length polymorphisms easily genotyped by fragment size differentiation (in similar fashion to widely established STR typing), whereas SNPs require determination of the polymorphic base through more complex direct or indirect sequencing methods. In brief, AIM-INDELs can offer the same potential as AIM-SNP assays for ancestry detection, but have the advantage of being very simply genotyped through a PCR followed by direct capillary electrophoresis of the amplified products - a system easily implemented by any laboratory with capillary analyzers. The simplicity of the INDEL approach delivers ease-of-use, time and cost effectiveness, and most important in forensic analysis, considerably reduces the steps involved in the genotyping of an ancestry-informative biallelic marker set in comparison with AIM-SNPs. The direct workflow minimizes manipulation, risks of contamination or sample mix-ups, and reduces to a minimum the number of variables affecting the end result. Furthermore, the direct fluorescence signals of INDEL alleles allow for mixture detection, providing a considerable additional benefit over AIM-SNPs assayed by SNaPshot.

In this study a set of 46 AIM-INDELs was selected to efficiently measure population admixture proportions of four different origins (African, European, East Asian and Native American). We have optimized the multiplexed genotyping of the 46 AIMs in a simple and informative assay, enabling a more straightforward alternative to AIM-SNP typing methods dependent on multi-step protocols or implementation of genotyping technologies that are expensive, complex and platform-dependent. In addition, we established reference databases using the HGDP-CEPH diversity panel samples [30] from the above four population groups and assessed the efficiency of the assay in inferring the ancestry of individuals from different test populations and estimating ancestry proportions at the individual and population level in an example admixed population.

## Materials and Methods

### Ethics Statement

The current study was approved by the Institute of Molecular Pathology and Immunology of the University of Porto institutional review board. Besides the HGDP-CEPH diversity panel human cell line samples, all other samples involved in the study are long-lasting anonymized DNA extracts previously obtained with informed written consent from healthy individuals for research purposes.

### Population samples

A total of 1002 DNA samples were used in this study comprising: i) reference samples from the HGDP-CEPH diversity panel standardized subset H952 [30], [31] with origin in Africa (AFR), Europe (EUR), East Asia (EAS), America (NAM) and also Oceania (OCE), representing a total 584 individuals from 40 populations. Individuals 1219, 1339, 1344 and 1041 were not included in the study since no DNA was available for analysis; in substitution of 1041 we used 1042 who had been excluded from subset H952 due to a parent/offspring relationship with 1041 [31]; ii) samples from Angola (48), Portugal (48), Taiwan (48) and Brazilian Amazonas tribes (48) used in a preliminary evaluation of the AIM-INDEL assay and as example testing samples; iii) samples from the city of Belém (226), an admixed population in northeastern Amazonas, Brazil.

### AIM-INDEL selection and development of the multiplex reaction

An initial pool of candidate INDELs was assembled by collecting previously available population data on this type of polymorphism included in the Marshfield Diallelic Insertion/Deletion Polymorphisms database website (http://www.marshfieldclinic.org/mgs/; [7]) and from later studies that also characterized some candidate INDELs in different population groups [27], [28], [32], [33]. Considering the allele frequency data compiled from the diverse sources, all markers were sorted according to frequency differentials (δ) [34] comparing four human population groups of Africans, Europeans, East Asians and Native Americans. For this study we selected a set of 46 markers (Table 1) among the most informative INDELs for each population group (all with δ≥0.40 between at least two groups) and optimized a unique multiplex reaction allowing the simultaneous genotyping of all AIMs in a single PCR and electrophoretic run. The multiplex development followed a similar workflow as in Pereira et al. [28], [35] except for the accommodation of certain longer amplicons into a broadened size window up to 230 bp in order to type an extended number of INDELs in a single reaction.

**Table 1:** AIM-INDELs used in the multiplex.

MID*	rs number	Chromosome	Position (bp)**	Alleles described in dbSNP	References
MID-1470	rs2307666	11	64729920	-/GTTAC	[7], [27], [33]
MID-777	rs1610863	16	6551830	-/GAA	[7]
MID-196	rs16635	6	99789775	-/CAT	[7], [27], [32]
MID-881	rs1610965	5	79746093	-/ACTT	[7], [32]
MID-3122	rs35451359	18	45110983	-/ATCT	[7]
MID-548	rs140837	6	3708909	-/CT	[7]
MID-659	rs1160893	2	224794577	-/CT	[7]
MID-2011	rs2308203	2	109401291	-/CTAGA	[7], [27]
MID-2929	rs33974167	8	87813725	-/TA	[7]
MID-593	rs1160852	6	137345857	-/TT	[7]
MID-798	rs1610884	5	56122323	-/GGGAAA	[7], [32]
MID-1193	rs2067280	5	89818959	-/AT	[7]
MID-1871	rs2308067	7	127291541	-/TT	[7]
MID-17	rs4183	3	3192524	-/TAAC	[7], [32]
MID-2538	rs3054057	15	86010538	-/AACA	[7]
MID-1644	rs2307840	1	36099090	-/GT	[7], [32]
MID-3854	rs60612424	6	84017514	-/TCTA	[7]
MID-2275	rs3033053	14	42554496	-/TCAGCAG	[7]
MID-94	rs16384	22	42045009	-/AAC	[7], [33]
MID-3072	rs34611875	18	67623917	-/GCCCCCA	[7]
MID-772	rs1610859	5	128317275	-/TAG	[7]
MID-2313	rs3045215	1	234740917	-/ATTATAACT	[7], [32]
MID-397	rs25621	6	139858158	-/TTCT	[7]
MID-1636	rs2307832	1	55590789	-/AA	[7], [32]
MID-51	rs16343	4	17635560	-/TTTAT	[7], [32]
MID-2431	rs3031979	8	73501951	-/ATTG	[7]
MID-2264	rs34122827	13	63778778	-/AAGT	[7]
MID-2256	rs133052	22	41042364	-/CAT	[7], [32]
MID-128	rs6490	12	108127168	-/ATT	[7]
MID-15	rs4181	2	42577803	-/AAATACACAC	[7], [32]
MID-2241	rs3030826	6	67176774	-/GTCCAATA	[7], [32]
MID-419	rs140708	6	170720016	-/AATGGCA	[7], [32]
MID-943	rs1611026	5	82545545	-/TGAT	[7]
MID-159	rs16438	20	25278470	-/CCCCA	[7]
MID-2005	rs2308161	10	69800909	-/AACAAT	[7], [33]
MID-250	rs16687	7	83887882	-/CA	[7], [32]
MID-1802	rs2307998	5	7814345	-/GGA	[7]
MID-1607	rs2307803	3	108981031	-/TG	[7]
MID-1734	rs2307930	6	84476378	-/CCAT	[7]
MID-406	rs25630	6	14734341	-/AG	[7]
MID-1386	rs2307582	1	247768775	-/AAACTATTCATTTTTCACCCT	[7], [27]
MID-1726	rs2307922	1	39896964	-/CAAGAACTATAAT/CACTATCTATTAT	[7], [27], [32]
MID-3626	rs11267926	15	45526069	-/AATATAATTTCTCCA	[7]
MID-360	rs25584	12	112145217	-/AA	[7]
MID-1603	rs2307799	5	70828427	-/TTGT	[7], [27], [33]
MID-2719	rs34541393	20	30701405	-/AACT	[7], [28]

*Nomenclature according to [7] and Marshfield Diallelic Insertion/Deletion Polymorphisms database;

**Mapping data according to dbSNP (build 132).

### Amplification and genotyping

PCR amplification of the 46 AIM-INDELs used the QIAGEN Multiplex PCR kit (Qiagen) at 1× Qiagen multiplex PCR master mix, 0.1 µM of all primers (sequence details in Table S1) and 0.3–5 ng of genomic DNA in a 10 µL final reaction volume. Thermocycling conditions were: initial step at 95°C for 15 min; 30 cycles at 94°C for 30 sec, 60°C for 90 sec, and 72°C for 45 sec; and a final extension at 72°C for 60 min. The PCR products were then prepared for capillary electrophoresis (CE) by adding 1 µL of amplified product to 10 µL Hi-Di™ Formamide (Applied Biosystems) and 0.3 µL of GeneScan™ 500 LIZ® size standard (Applied Biosystems). CE was performed using a 3130 Genetic Analyzer prepared with DS-33 matrix standard, POP-7™ polymer and applying virtual filter G5 (Applied Biosystems). The electropherograms were analyzed and genotypes were automatically assigned with GeneMapper v4.0 (Applied Biosystems). For practical reasons INDEL short alleles were coded as 1 and long alleles as 2.

### Statistical analysis

Estimation of allele frequencies, exact tests of Hardy-Weinberg equilibrium (HWE), F_ST_ genetic distances and linkage disequilibrium tests were assessed using Arlequin v3.5.1.2 [36]. Ancestry inferences were performed using STRUCTURE v2.3.3 [17], [37] with a burnin length of 100,000 followed by 100,000 MCMC repetitions and a variety of parameter sets were tested depending on the objective of the analysis. Initial runs were made without any prior information on the origin of samples, using the “Admixture Model” and considering either correlated or independent “Allele Frequency Models”; a minimum of 3 independent runs were performed for each testing K value, ranging from K = 1 to K = number of presumed clusters present in the dataset plus three. The estimated ln probability of data (−lnP(D)) values were plotted using Structure harvester v0.6.6. (http://taylor0.biology.ucla.edu/structureHarvester/). In a second phase, when using reference samples as training sets to test for “unknown” individuals or populations, STRUCTURE analyses were carried out using the same parameters as before or selecting the “Use Population Information” option. In these cases, allele frequencies were updated using only the reference individuals with POPFLAG = 1 data (option under the Advanced tab). Here, 3 independent runs were performed only for the appropriate number of clusters, as evaluated by the initial analysis. Unless otherwise indicated, results are presented for the default settings considering the “Admixture Model” and correlated allele frequencies. CLUMPP v1.1.2 [38] was used to obtain the average permutated individual and population Q-matrices throughout the three replicates for each K value. Those matrices were used as input to *distruct* v1.1 [39] to obtain bar plots where each individual is represented as a segment divided into K colors that represent the estimated membership coefficients from each cluster.

Principal component analysis (PCA) was performed as an additional and independent approach to estimate the number of populations present in the data set. We used R 2.11.1 [40] with SNPassoc package [41] to obtain two and three dimensional graphics and the information percentage values associated to each principal component.

The efficiency of the 46 AIM-INDEL set for assigning individuals to population groups was further evaluated by one-out cross-validation based on a flexible single profile analysis system very similar to STRUCTURE, calculating likelihood ratio values obtained with a Bayesian classification algorithm implemented in the “Snipper app suite” website (http://mathgene.usc.es/snipper/; [23]).

## Results

**Figure 1:**
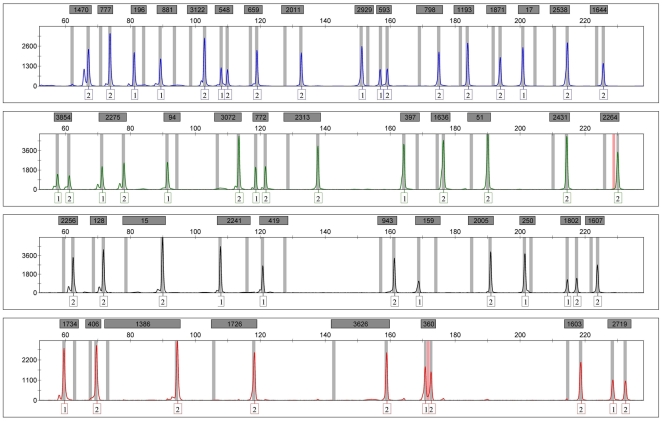
Example of an electropherogram obtained for the HGDP-CEPH 0452 sample with the 46 AIM-INDEL multiplex (markers are identified by MID number).

A simple and informative multiplex was developed for the simultaneous analysis of 46 AIM-INDELs reported to have high δ values between the AFR, EUR, EAS or NAM population groups. All markers were analyzed in short fragments (<230 bp) through a single PCR followed by capillary electrophoresis (Figure 1). The workflow of the INDEL assay is straightforward, reducing considerably the steps and resources needed to genotype a large set of biallelic AIMs.

After optimization of the method we created a database including HGDP-CEPH diversity panel genetic data, commonly used by the research community as reference populations for the four groups AFR, EUR, EAS, NAM and also from Oceania (complete database included in File S1).

### Genetic characterization of reference populations

Patterns of INDEL variability observed in the HGDP-CEPH samples from the population groups AFR, EUR, EAS and NAM are detailed in Table S2 as well as δ and pairwise F_ST_ for each marker. With few exceptions, the vast majority of the INDELs show high allele frequency differentials and genetic distances between at least two groups (39 with δ≥0.4 and 44 with δ≥0.3). No significant departures from Hardy-Weinberg equilibrium were found in the studied populations and pairwise linkage disequilibrium exact tests did not detect significant associations within the marker set.

One interesting finding was the occurrence of an unexpected third allelic state (coded as allele 3) for MID360 and MID2264. Sequencing analysis confirmed our observations as a result of additional sequence length variants within the amplicon fragments. For MID360 the third allelic state observed is due to a T insertion associated with the short allele, 8 bases downstream of the targeted polymorphism (allele 1D8Tins). Conversely for MID2264, allele 3 corresponds to a T deletion occurring in the long allele background (allele 2D68Tdel). Interestingly, the MID360 variant alleles were only found in AFR samples whereas the MID2264 variants seemed specific of EUR, further contributing to the differentiation of the two groups.

### Inferring genetic ancestry

#### - The AIM-INDEL panel efficiently distinguishes four major population groups

Before implementing the HGDP-CEPH diversity panel reference database, a preliminary evaluation on the performance of a panel comprising 44 INDELs at the time (without MID94 and MID1734) had been performed using 48 samples with origin in each of the four groups under study (detailed results in Figure S1). In brief, analyses with STRUCTURE, PCA and one-out cross validation clearly supported the efficiency of the panel in clustering individuals into four population groups.

**Figure 2:**
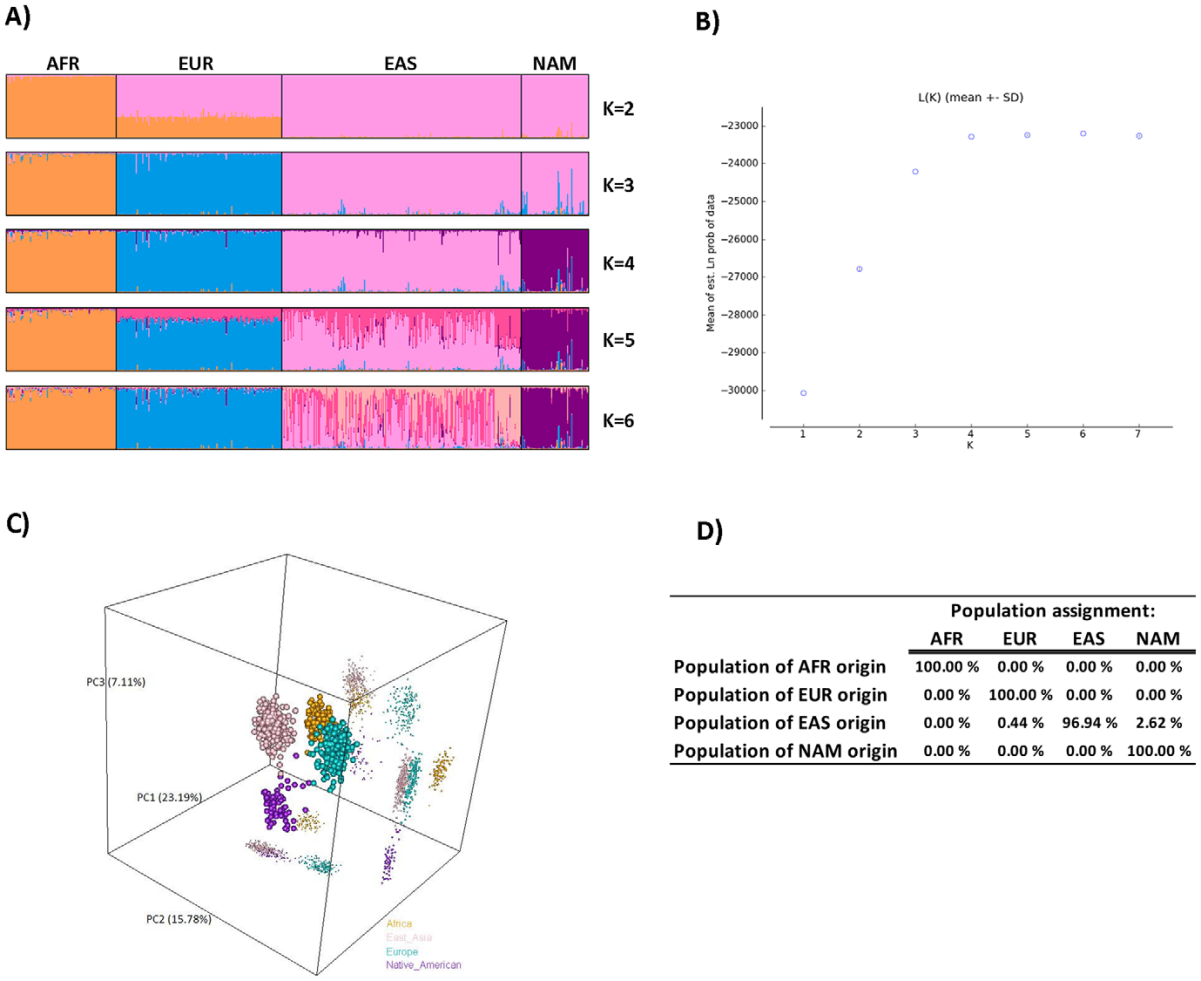
Analysis of HGDP-CEPH diversity panel samples from four continental origins using a set of 46 AIM-INDELs. A) ancestral membership proportions (based on STRUCTURE results from 3 independent runs treated in CLUMPP and plotted with *distruct*; individuals were first sorted by geographic origin of population, and within those by ascending population code and HGDP individual number); B) estimated ln probability of the data (−ln P(D) obtained with STRUCTURE and plotted using Structure harvester); C) principal component analysis 3D plots. D) estimation of population assignment success (results from one-out cross validation studies using the Snipper app suite; see methods for details of the analyses). AFR: Africa; EUR: Europe; EAS: East Asia; NAM: Native America.

The results obtained for the complete AIM-INDEL panel with HGDP-CEPH AFR, EUR, EAS and NAM populations strongly corroborate these preliminary findings (Figure 2). STRUCTURE ancestry estimates considering K = 4 still produce an enhancement in −ln P(D) values while a plateau is reached thereafter, which points to 4 as the smallest K number capturing the major population structure in the data and supports the inference that a four group clustering better fits the genetic data (Figure 2A and 2B).

PCA for the same dataset allows an independent non-model based view of the individual clustering. The first three PCs define approximately half of the variance in the dataset (46.1%) yet allow a clear spatial separation of four different groups (Figure 2C). Likewise, cross-validation studies (Figure 2D) revealed the INDEL panel to show a high accuracy of population assignment, with a global classification error of 1.26% (specifically 7 of 556). All AFR, EUR and NAM were correctly assigned whereas misclassified individuals were all from the Yakut population in Siberia except for one individual from Oroquen, China.

#### - HGDP-CEPH genetic data as reference genotypes to test individuals or populations of unknown origin

**Figure 3:**
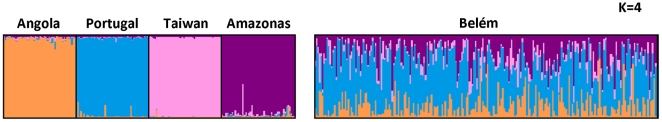
Ancestral membership proportions for testing population samples from different continental origins using the HGDP-CEPH diversity panel genetic data as training sets. Angola (Africa); Portugal (Europe); Taiwan (East Asia); Brazilian Amazonas tribes (Native America); Belém is an example of a highly admixed Brazilian city in northeastern Amazonas.

Reference HGDP-CEPH diversity panel genetic data from the four population groups (AFR, EUR, EAS and NAM) was used to estimate ancestry proportions of individuals/populations from different geographic locations. We tested samples from Angola, Portugal, Taiwan and Brazil (Amazonas Amerindian tribes and Belém, a northeastern Amazonas city). The individual and global admixture estimates obtained with genetic data only (no prior population information) correspond well with expected patterns, knowing the origin of the subjects (Figure 3; Table 2). In general, individuals from the non-admixed populations show high membership proportion in the same cluster as HGDP-CEPH representatives of the same population group. In contrast individuals from Belém show highly variable admixture patterns mainly of European, Native American and African origin (Figure 3; Table 2), resulting in average ancestry proportions of 53.5% EUR, 22.9% NAM, 14.8% AFR and 8.8% EAS. Considering the historical formation and peopling of Brazil in which there were three main contributing ancestral populations (NAM, EUR and AFR) we performed a three-group analysis for the particular case of Belém – specifically, excluding EAS and using only NAM, EUR and AFR ancestral groups with K = 3 (Figure S2). In particular the Native American proportion increased (53.7% EUR, 29.5% NAM and 16.8% AFR), having captured most of the previous East Asian component.

**Table 2:** Ancestral membership proportions for HGDP-CEPH diversity panel samples and testing populations from four continental origins.

	46 AIM-INDELs (this study)				210 INDELs [32]				48 I_n_4 AIM-SNP set [44]				
	AFR	EUR	EAS	NAM	AFR	EUR	EAS	NAM		AFR	EURA	EAS	AMI
HGDP-CEPH AFR	**0.969**	0.011	0.012	0.008	**0.977**	0.009	0.009	0.005	AFR	**0.97**	0.02	0.01	0.01
HGDP-CEPH EUR	0.008	**0.963**	0.014	0.014	0.007	**0.967**	0.013	0.013	EURA	0.01	**0.96**	0.02	0.01
HGDP-CEPH EAS	0.006	0.018	**0.952**	0.024	0.007	0.021	**0.955**	0.017	EAS	0.01	0.04	**0.91**	0.03
HGDP-CEPH NAM	0.008	0.041	0.027	**0.924**	0.011	0.028	0.015	**0.946**	AMI	0.01	0.03	0.04	**0.92**
Testing populations:	AFR	EUR	EAS	NAM									
Angola	**0.970**	0.011	0.011	0.008									
Portugal	0.018	**0.966**	0.008	0.008									
Taiwan	0.004	0.003	**0.984**	0.009									
Br. Amazonas tribes	0.010	0.013	0.032	**0.945**									
Belém (4G-analysis)	0.148	0.535	0.088	0.229									
Belém (3G-analysis)	0.168	0.537	-	0.295									

(AFR: Africa; EUR: Europe; EAS: East Asia; NAM: Native America).

#### - Indications of population differentiations beyond four groups from inclusion of Oceanians

**Figure 4:**
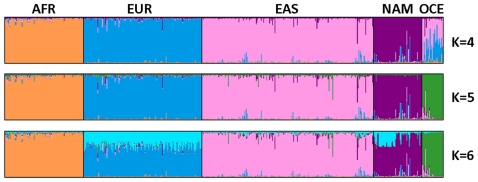
Ancestral membership proportions for HGDP-CEPH diversity panel samples from five continental origins using a set of 46 AIM-INDELs (based on STRUCTURE results from 3 independent runs treated in CLUMPP and plotted with *distruct*; individuals were first sorted by geographic origin of population, and within those by ascending population code and HGDP individual number). AFR: Africa; EUR: Europe; EAS: East Asia; NAM: Native America; OCE: Oceania.

The AIM-INDEL panel was primarily designed as a tool for ascertaining ancestry from four major population groups. Nonetheless, as there is general interest in AIM panels able to distinguish populations at the broader continental level, we extended our study to HGDP-CEPH Oceanian samples and assessed the ability of the panel to differentiate populations with origin in all five continent regions. Following the same evaluation strategy as before, the assay proved to consistently recognize a fifth cluster corresponding to Oceanians and that K = 5 captures most of the structure in the dataset (Figure 4; Figure S3 for details). PCA plots (Figure S3C) show most HGDP-CEPH Oceanians form a distinguishable cloud lying between EUR and EAS even though the separation is not perfectly achieved. In a five-group classification, the one-out cross validation error rate increased slightly to 1.54% (9/584). The assignment of Oceanians was accurately made but two EAS (from Cambodia) were now misclassified as OCE.

## Discussion

The main objective of this study was to provide a simple tool for inferring ancestry and estimate admixture proportions from four different population origins that can be widely applied to genetic studies. We describe a new AIM assay comprising 46 INDELs that are simply analyzed in a multiplex PCR followed by CE detection. With this approach we were able to combine the ancestry informative power of biallelic markers (exemplified by AIM-SNP panels) with the simplified analysis based in fragment size separation (as in STR typing). The methodology of the assay is straightforward and can be readily and inexpensively implemented in any molecular genetics laboratory. In contrast, the majority of AIM sets published in recent years involve more complex genotyping protocols or are limited to specific platforms not available to all laboratories and therefore requiring additional resources [e.g. 23], [27], [42]–[49]. Another important aspect is that some AIM sets are directed to differentiate specific population groups depending on the main ancestral contributors to the individuals or populations under investigation [e.g. 23], [27], [50]. We aimed to develop a generic panel, designed to target the four major population groups of AFR, EUR, EAS and NAM, similarly to Halder et al. [43]. Our objective was to balance combining the highest number of AIMs possible into a single reaction with use of amplicon lengths suitable for the analysis of low quality DNA. The limitation of large multiplex reliability restricts the maximum number of markers in a single reaction. On the other hand, AIMs have an important application in forensic investigations where the quantity and quality of the samples are often limiting factors. We were eventually able to multiplex 46 highly informative INDELs, with a scope of markers comparable to other AIM sets reported [23], [27], [46]–[48]. Kosoy et al. [44] have shown that small AIM sets can distinguish major population groups and correct for false positive results in association studies. Other studies have addressed ancestry prediction of the HGDP-CEPH samples using large-scale SNP datasets obtained with high-throughput microarrays, and have also evaluated the performance of small subsets of markers ascertained following different strategies such as F_ST_, allele differentials ä, informativeness of assignment index I_n_ [51] or PCA (e.g. [42], [48], [52]). These studies have shown that inference of continental ancestry for the HGDP-CEPH panel is quite clear, and can be performed with a relatively small number of SNPs (10 to 50). They also showed that, when using SNPs, it is possible to predict individual ancestry down to the population level, although such approaches require an increased number of markers ranging from several hundred to thousands [48], [52]. Due to the multiplexing limitation associated with the number of markers that it is possible to analyze in a single PCR, and in the same respect for other small-scale AIM sets, the 46 AIM-INDEL assay we outline is only going to be particularly useful when broad assignment to continental ancestry is desired, or when estimating admixture proportions in individuals/populations that received ancestral contributions of different continental origins. Assessing within-continent population structure requires much larger arrays of markers, well beyond the number included in our set and in most of the alternative AIM sets, and therefore it will have limited application for that purpose.

The AIM-INDEL assay allowed a rapid and cost-effective genotyping of a large number of samples including HGDP-CEPH individuals from five continental groups (AFR, EUR, EAS, NAM and OCE) and representative testing samples with different origins and admixture levels. From the genetic characterization of the reference ancestral samples we observed a high level of differentiation from the chosen INDELS, as expected from the selection criteria. Although some markers revealed lower differences than those expected from previous data, this is possibly due to the samples representing each group and allele frequency estimation strategies being different [e.g. 7]. The pairwise F_ST_ values obtained with the 46 AIM-INDELs (Table S2) are clearly above the usually found at the continental level with random markers [45], [53] and similar to those obtained with other AIM panels for the same population groups [44]–[46].

The results from the HGDP-CEPH diversity panel and other representative populations underlined the capacity of the panel to distinguishing four continental population groups. Furthermore, the ancestry estimates obtained in a four-group analysis are very similar to those obtained in Kosoy et al. [44] with a 48 I_n_4 AIM-SNP set for equivalent population groups (Table 2), as well as using a much larger number of INDELs (210) for the same HGDP-CEPH individuals (Table 2; [32]). This concordance in the ancestry estimates highlights the accuracy of the AIM-INDEL panel in inferring ancestry proportions from African, European, East Asian and Native American origin. Furthermore, in spite of the assay being primarily designed for studies considering only four major population groups, extension to five groups revealed the capacity to reliably distinguish Oceanians.

The population assignment cross validation studies based on Bayesian likelihood ratios provided additional evidence of the utility of the assay, particularly for forensic applications where single profiles are often analyzed one at a time. Here the error rates in classifications considering either four or five population groups were low (1.26% and 1.54% respectively). The AIM-INDEL panel achieves very high accuracy for population assignment in the five broad continental regions, similar to results observed by Paschou et al. when using subsets of 50 SNPs ascertained by PCA and estimation of I_n_ metrics [48]. In our study, the great majority of misclassified individuals were from a single population (Yakut of eastern Siberia) localized near the northeastern fringe of the Asian continent. This intermediate position between East Asia and the American continent can explain differences in patterns of divergence between individuals and their misclassification as American. Likewise, the cross validation studies with five groups revealed two misclassified Cambodians as Oceanians. Together, these results suggest a weaker performance of the panel with differentiation of East Asians. In fact, the accumulated divergence assessed for EAS *vs.* others is slightly smaller than for the other ancestral groups, and the fact that the HGDP-CEPH EAS group analyzed is so diverse (229 individuals from 18 subpopulations) may contribute to this reduced differentiation for East Asians. Another important aspect is the proximity of the East Asian and Native American gene pools. Considering the history of modern humans these groups have diverged over the shortest time, and furthermore, the original peopling of Americas from Beringia involved a significant bottleneck effect that is still reflected in Native American variability. Despite this slightly reduced level of differentiation in the AIM-INDELs selected, STRUCTURE, PCA and cross validation studies together support the capacity of the panel to properly distinguish both groups.

AIM panels are regularly applied in population genetics studies to analyze admixed populations by estimating admixture proportions both at the individual and population level. Depending on the historical context of populations under study, there are different principal ancestral contributors to the formation of the current ancestry characteristics of the region. For example, Brazil and the majority of south-American countries underwent admixture between the pre-existent Native Americans, colonizing Europeans and later African influences resulting from the slave trade to create essentially tri-hybrid populations. In such cases, it is appropriate for genetic studies to perform three-group analyses of ancestry estimates. Our study analyzed ancestry proportions in Belém. We first considered the possibility of a fourth EAS minor ancestral contributor in initial analyses and K = 4 resulted in a low level but detectable fraction of membership of this cluster at 8.8% (Table 2). However, although not statistically significant (exact test of differentiation *p* value = 0.136), the three-group membership proportion estimates at K = 3 showed a noticeable increase in the Native American component to 29.5% (Table 2) which is in very close agreement with the admixture proportions previously reported for the same population but using a different set of AIM-INDELs (average NAM estimate: 28.4%; [27]). Nevertheless, a preliminary four-group analysis has persuasive arguments for considering all four potential contributors to admixture in these regions. In particular, some locations in Brazil (e.g. São Paulo, Campinas; IBGE – *Instituto Brasileiro de Geografia e Estatística*, www.ibge.gov.br) include significant East Asian communities, despite having joined these populations rather recently. When using Brazilian samples from such geographic areas, particularly as case and control samples for association studies, a preliminary four-group analysis is recommended to detect the presence of East Asian ancestry amongst individuals in the study. Otherwise there is considerable risk that the *a priori* rejection of this hypothesis based on three-group analyses could lead to an over-estimation of the Native American proportion in the global admixture estimates (data not shown) due to a strong bias caused by the presence of East Asian individuals in the population under study. Conversely, when “forcing” a four-group analysis in south-American tri-hybrid populations, it is possible that the fourth East Asian component can produce a spurious fraction of membership arising from the Native American component, due to the close relationship of the East Asian and Native American population groups. In summary, we advocate adopting an approach taking due regard for the particular population under study. Consideration of the known recent population history and demographics helps make appropriate adjustment for the different principal ancestral contributors. In the special case of south-American populations, we recommend a preliminary study taking advantage of the full potential of the AIM-INDEL assay to identify and possibly exclude East Asian study subjects, and subsequently perform a comprehensive three-group analysis. The AIM-INDEL assay can be efficiently used in three-group analyses AFR/EUR/NAM, similarly to [27] and also AFR/EUR/EAS, as in [23]. Nonetheless, the reliability of the four-way analysis we repeatedly achieve with this multiplex allows a clear distinction of all groups.

In conclusion, we have optimized the multiplexed genotyping of 46 AIM-INDELs in a simple and informative assay, enabling a more straightforward alternative to the commonly available AIM-SNP typing methods dependent on multi-step protocols and/or implementation of dedicated genotyping technologies. The AIM-INDEL assay produces accurate individual ancestry estimates of four different origins, which can be applied to the correction of false positive results due to population stratification between case and control samples in association studies. Most effectively it can be used as a simple and inexpensive tool for the initial screening of individuals prior to expensive GWA studies or to allow precise matching of ancestries amongst case and control samples. Finally, given the relatively high efficiency in population assignment of individuals from all five continental origins, the multiplex represents a tool of considerable potential in forensic applications.

## Supporting Information

Figure S1 **Analysis of population samples from four different continental origins using a preliminary set of 44 AIM-INDELs (without MID94 and MID1734).** A) ancestral membership proportions (based on STRUCTURE results from 3 independent runs treated in CLUMPP and plotted with distruct); B) estimated ln probability of the data (−lnP(D) obtained with STRUCTURE and plotted using Structure harvester); C) principal component analysis 3D plots; D) estimation on population assignment success (results from one-out cross validation studies using the Snipper app suite; see methods for details on the analyses). Angola (Africa); Portugal (Europe); Taiwan (East Asia); Brazilian Amazonas tribes (Native America).
(PDF)

Figure S2 **Ancestral membership proportions in the Brazilian city of Belém using HGDP-CEPH diversity panel genetic data of three main ancestral contributors as training sets.** A) bar plots based on STRUCTURE results from 3 independent runs treated in CLUMPP and plotted with distruct (AFR: Africa; EUR: Europe; NAM: Native America); B) triangular plots based on STRUCTURE results from the run with highest −lnP(D) (left: admixture model; right: using population information; red: Africa; green: Europe; blue: Native American; yellow: Belém).
(PDF)

Figure S3 **Analysis of HGDP-CEPH diversity panel samples from five continental origins using a set of 46 AIM-INDELs.** A) ancestry membership proportions (estimated based on STRUCTURE results from 3 independent runs treated in CLUMPP and plotted with distruct; individuals were first sorted by geographic origin of population. and within those by ascending population code and HGDP individual number); B) estimated ln probability of the data (−lnP(D) obtained with STRUCTURE and plotted using Structure harvester); C) principal component analysis 3D plots. D) estimation on population assignment success (results from one-out cross validation studies using the Snipper app suite; see methods for details on the analyses). AFR: Africa; EUR: Europe; EAS: East Asia; NAM: Native America; OCE: Oceania.
(PDF)

Table S1 **PCR primer sequences used in the multiplex.**
(PDF)

Table S2 **Allele frequencies, δ and FST values for the 46 AIM-INDELs in HGDP-CEPH diversity panel population samples from Africa (AFR), Europe (EUR), East Asia (EAS) and Native America (NAM).**
(PDF)

File S1 **Genotypic data (STRUCTURE format) for the 46 AIM-INDELs in HGDP-CEPH diversity panel population samples from Africa, Europe, East Asia and Native America.**
(TXT)
