# Supplementary material for: Straightforward Inference of Ancestry and Admixture Proportions through Ancestry-Informative Insertion Deletion Multiplexing
Source: PLoS One. 2012 Jan 17;7(1):e29684. doi: 10.1371/journal.pone.0029684 (PMC3260179; doi:10.1371/journal.pone.0029684)
Supplement: Figure S1 — Analysis of population samples from four different continental origins using a preliminary set of 44 AIM-INDELs (without MID94 and MID1734). A) ancestral membership proportions (based on STRUCTURE results from 3 independent runs treated in CLUMPP and plotted with distruct); B) estimated ln probability of the data (−lnP(D) obtained with STRUCTURE and plotted using Structure harvester); C) principal component analysis 3D plots; D) estimation on population assignment success (results from one-out cross validation studies using the Snipper app suite; see methods for details on the analyses). Angola (Africa); Portugal (Europe); Taiwan (East Asia); Brazilian Amazonas tribes (Native America). (PDF) [file pone.0029684.s001.pdf]

A)

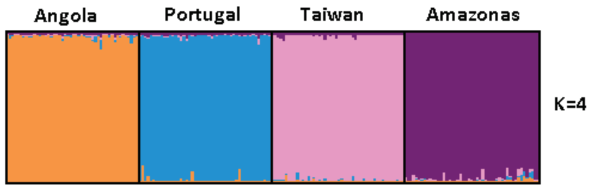

B)

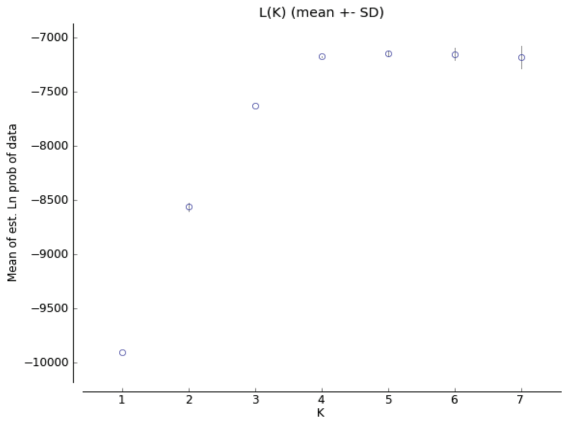

C)

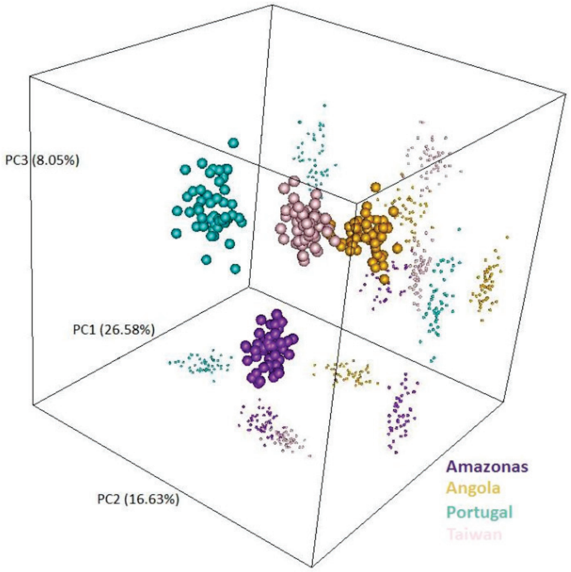

D)

	Population assignment:			
	Angola	Portugal	Taiwan	Amazonas
**Population of Angola origin**	100.00 %	0.00 %	0.00 %	0.00 %
**Population of Portugal origin**	0.00 %	100.00 %	0.00 %	0.00 %
**Population of Taiwan origin**	0.00 %	0.00 %	100.00 %	0.00 %
**Population of Amazonas origin**	0.00 %	0.00 %	0.00 %	100.00 %

Figure S1 Analysis of population samples from four different continental origins using a preliminary set of 44 AIM-INDELs (without MID94 and MID1734): A) ancestral membership proportions (based on STRUCTURE results from 3 independent runs treated in CLUMPP and plotted with *distruct*); B) estimated ln probability of the data (-lnP(D) obtained with STRUCTURE and plotted using Structure harvester); C) principal component analysis 3D plots; D) estimation on population assignment success (results from one-out cross validation studies using the Snipper app suite; see methods for details on the analyses). Angola (Africa); Portugal (Europe); Taiwan (East Asia); Brazilian Amazonas tribes (Native America).
