# Supplementary material for: Straightforward Inference of Ancestry and Admixture Proportions through Ancestry-Informative Insertion Deletion Multiplexing
Source: PLoS One. 2012 Jan 17;7(1):e29684. doi: 10.1371/journal.pone.0029684 (PMC3260179; doi:10.1371/journal.pone.0029684)
Supplement: Figure S2 — Ancestral membership proportions in the Brazilian city of Belém using HGDP-CEPH diversity panel genetic data of three main ancestral contributors as training sets. A) bar plots based on STRUCTURE results from 3 independent runs treated in CLUMPP and plotted with distruct (AFR: Africa; EUR: Europe; NAM: Native America); B) triangular plots based on STRUCTURE results from the run with highest −lnP(D) (left: admixture model; right: using population information; red: Africa; green: Europe; blue: Native American; yellow: Belém). (PDF) [file pone.0029684.s002.pdf]

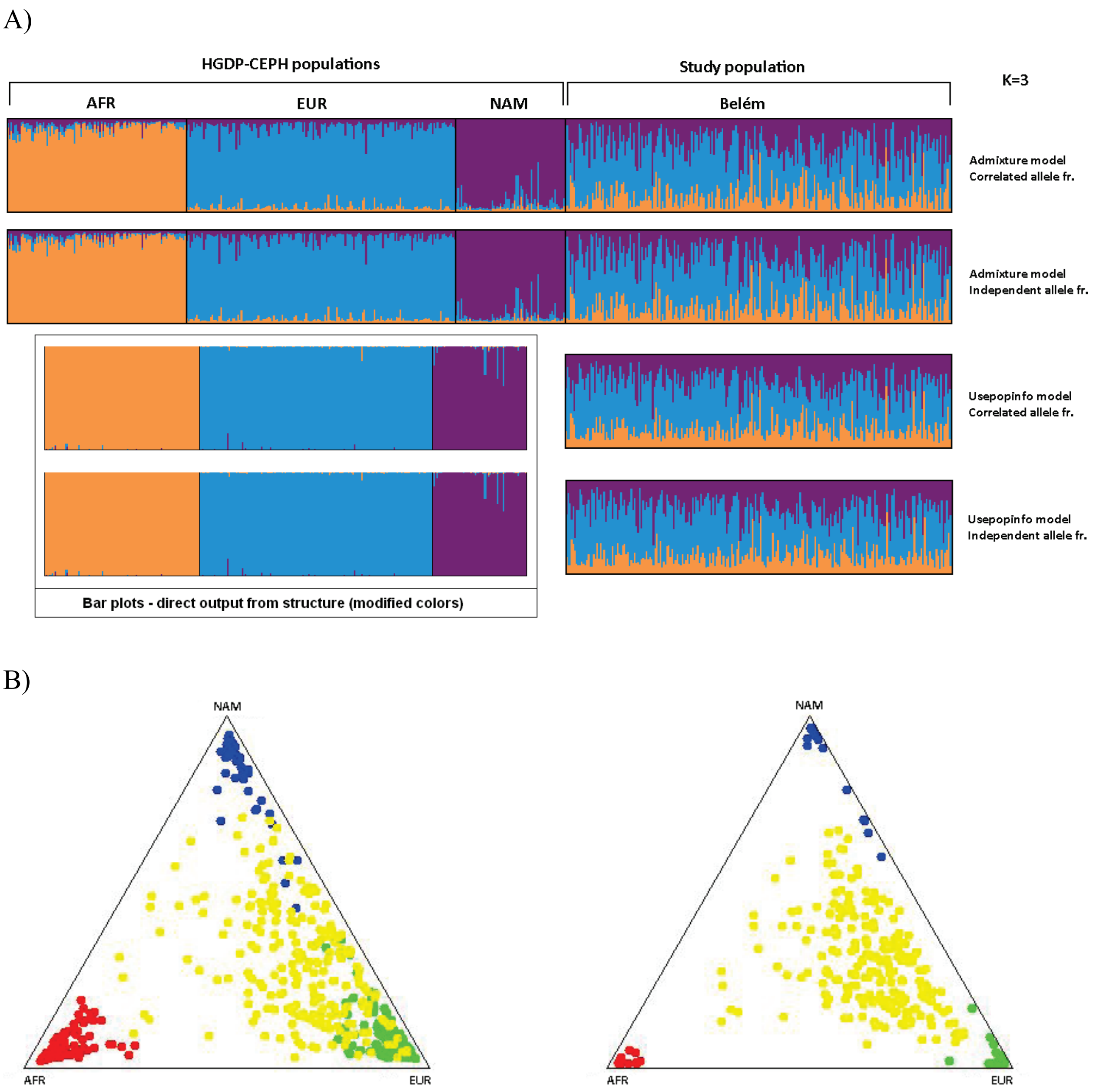


**Figure S2** Ancestral membership proportions in the Brazilian city of Belém using HGDP-CEPH diversity panel genetic data of three main ancestral contributors as training sets: A) bar plots based on STRUCTURE results from 3 independent runs treated in CLUMPP and plotted with *distruct* (AFR: Africa; EUR: Europe; NAM: Native America); B) triangular plots based on STRUCTURE results from the run with highest -lnP(D) (left: admixture model; right: using population information; red: Africa; green: Europe; blue: Native American; yellow: Belém).
