# Supplementary material for: Straightforward Inference of Ancestry and Admixture Proportions through Ancestry-Informative Insertion Deletion Multiplexing
Source: PLoS One. 2012 Jan 17;7(1):e29684. doi: 10.1371/journal.pone.0029684 (PMC3260179; doi:10.1371/journal.pone.0029684)
Supplement: Figure S3 — Analysis of HGDP-CEPH diversity panel samples from five continental origins using a set of 46 AIM-INDELs. A) ancestry membership proportions (estimated based on STRUCTURE results from 3 independent runs treated in CLUMPP and plotted with distruct; individuals were first sorted by geographic origin of population. and within those by ascending population code and HGDP individual number); B) estimated ln probability of the data (−lnP(D) obtained with STRUCTURE and plotted using Structure harvester); C) principal component analysis 3D plots. D) estimation on population assignment success (results from one-out cross validation studies using the Snipper app suite; see methods for details on the analyses). AFR: Africa; EUR: Europe; EAS: East Asia; NAM: Native America; OCE: Oceania. (PDF) [file pone.0029684.s003.pdf]

A) B)

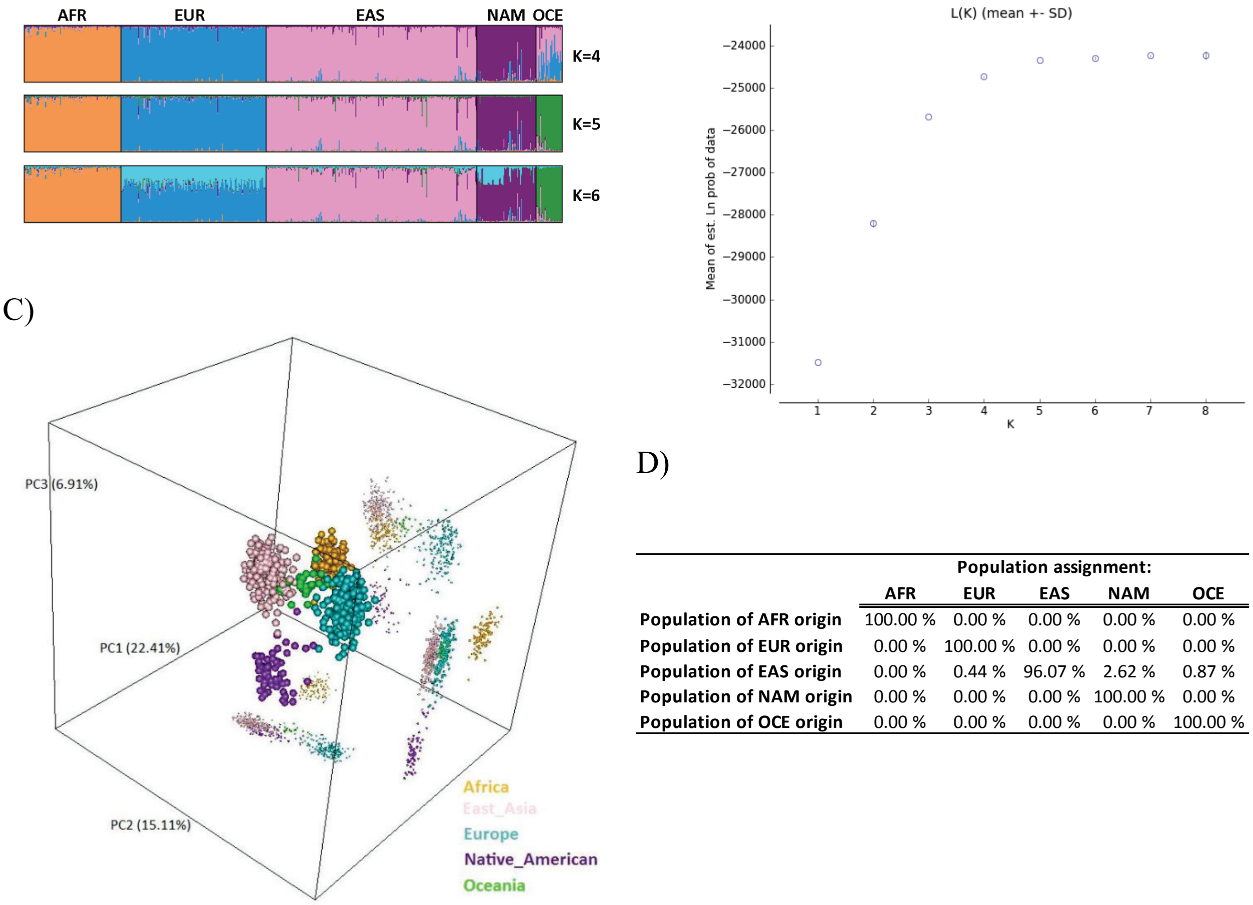

D)

	Population assignment:				
	AFR	EUR	EAS	NAM	OCE
**Population of AFR origin**	100.00 %	0.00 %	0.00 %	0.00 %	0.00 %
**Population of EUR origin**	0.00 %	100.00 %	0.00 %	0.00 %	0.00 %
**Population of EAS origin**	0.00 %	0.44 %	96.07 %	2.62 %	0.87 %
**Population of NAM origin**	0.00 %	0.00 %	0.00 %	100.00 %	0.00 %
**Population of OCE origin**	0.00 %	0.00 %	0.00 %	0.00 %	100.00 %

Figure S3 Analysis of HGDP-CEPH diversity panel samples from five continental origins using a set of 46 AIM-INDELs: A) ancestry membership proportions (estimated based on STRUCTURE results from 3 independent runs treated in CLUMPP and plotted with *distruct*; individuals were first sorted by geographic origin of population. and within those by ascending population code and HGDP individual number); B) estimated ln probability of the data (-lnP(D) obtained with STRUCTURE and plotted using Structure harvester); C) principal component analysis 3D plots. D) estimation on population assignment success (results from one-out cross validation studies using the Snipper app suite; see methods for details on the analyses). AFR: Africa; EUR: Europe; EAS: East Asia; NAM: Native America; OCE: Oceania.
