# Supplementary material for: Straightforward Inference of Ancestry and Admixture Proportions through Ancestry-Informative Insertion Deletion Multiplexing
Source: PLoS One. 2012 Jan 17;7(1):e29684. doi: 10.1371/journal.pone.0029684 (PMC3260179; doi:10.1371/journal.pone.0029684)
Supplement: Table S1 — PCR primer sequences used in the multiplex. (PDF) [file pone.0029684.s004.pdf]

Table S1 PCR primer sequences used in the multiplex

MID	rs number	Labeled primer	Unlabeled primer*
MID-1470	rs2307666	6FAM-GAGTCTGACCCTTCATAAGC	*g*CCATGGTGATATTACGTCCC
MID-777	rs1610863	6FAM-TGGAAGACACGTCCTAAGAG	*g*TATTCCTCCAGGCTCTTTGC
MID-196	rs16635	6FAM-CCAAGTTCTAGCCATATGGA	*gttctt*GACTATCTTCTCTGACCATC
MID-881	rs1610965	6FAM-TTGGCTCCCTATGATAATCC	*gtttc*TTGTGTTCCCAAAGTTCTCC
MID-3122	rs35451359	6FAM-TCACAAGTCCGGAATACCAG	*g*AGTTATGGGATGGGAAGGAG
MID-548	rs140837	6FAM-AGTCAGGACTGAAGAAACCC	*gttt*CAGTAAAACAAGAGCCCGTG
MID-659	rs1160893	6FAM-CACTGCATCAGACTGACTTC	*gtttctt*GGCTGCTTTGCTTTGAATTG
MID-2011	rs2308203	6FAM-TGAGAAACTAGGAGCTCTGG	*gt*TTCCTAAGAGCCACTGACAT
MID-2929	rs33974167	6FAM-TGTGATGTGGATAGGCAAGG	*g*AGGCTCCATTTGTTAAGAGG
MID-593	rs1160852	6FAM-TGCTCACTTTAGTGGAGACC	*gt*TTGCGTTTAGGTCCCTTCTG
MID-798	rs1610884	6FAM-ACGACAGTGTTCACAAAGAG	*g*CTGTTGTCTGACCTGTGAAG
MID-1193	rs2067280	6FAM-GCTGGGTAGTTTTTTCCTCC	*gt*TCCACCATCTACCTTCTATG
MID-1871	rs2308067	6FAM-TTGTAGTCAGAGAGTGTGCC	*g*AGCCTTTTCCCTAACGTCAC
MID-17	rs4183	6FAM-AGAAACTGCAACCCTCCAAG	GATCCCAGACACTGAAGATG
MID-2538	rs3054057	6FAM-CTCGCAAAGTAGGCAAGTTC	*g*ACACCAACAATCTTGGCACC
MID-1644	rs2307840	6FAM-ACACCACTGAAGATCTGACC	GGTCTAAAGTCAGTGCACAG
MID-3854	rs60612424	VIC-TCACCCTTATTCAGGGTTGC	GCCAGGGATTTAGTGTAGAG
MID-2275	rs3033053	VIC-CTACCTGACTACCACCTATG	*g*ACCCAGCCTATCTGACTTTG
MID-94	rs16384	VIC-TGGTGGCTCATGCACTTTTG	*gttt*ACAGGGTCTCGCTATGATGC
MID-3072	rs34611875	VIC-AGCTTTTTCCGGCAACTCTC	*gtt*TGGATGTGTCTGAGCTCAAC
MID-772	rs1610859	VIC-GTCTCRTTTTCCTGCAGTAG	*gtttctt*ATCCTTCTGCTCACTCTACC
MID-2313	rs3045215	VIC-GCACACATGCAGAAATGCAG	GTTGTAACATCTGTGAGGTC
MID-397	rs25621	VIC-TGGGCTTCTTCTGGGAAAAC	*g*CCACATTCAGGCGTTTTGTC
MID-1636	rs2307832	VIC-TTAGGAAGAGGTGCTATGGG	*g*CCTCCTTTGAAGACACACAG
MID-51	rs16343	VIC-AAGATTGGAGGGAAAAGTGC	*g*CGTCCTCCACCTTCTTTTTC
MID-2431	rs3031979	VIC-AGGAGGAGCTGATAGACTTC	GCAGTGTTGCAACTGATACG
MID-2264	rs34122827	VIC-CTTTGGCTATCCTGTCTCAC	GTAGGAGACCACTCACATTC
MID-2256	rs133052	NED-ATCGAACCGTTCCTAAGGAC	GCAAGAAAAGGAATCCAGGC
MID-128	rs6490	NED-ATCAGGAGACAATCCAGCAG	*g*TCCAGCCATTCAGACAAAGG
MID-15	rs4181	NED-GGGTTATTTGCCTCATCTCC	*gtttct*AGGTATTCTCTGTTCCCACG
MID-2241	rs3030826	NED-ACATACACGTGGAAGACTGC	*gtt*ACTGTCGACTGATCCAATAG
MID-419	rs140708	NED-CAGGAAAGTATGGCCCATTC	GTCCATGTTTTCTTTGAGCATC
MID-943	rs1611026	NED-TCTTCCTACCCCTGTTAGTG	GACAAGATCACTAGCTTGAC
MID-159	rs16438	NED-ACCAGAGCACTACAGCCTTT	*g*CAAGGYAGTAACAATGAGGG
MID-2005	rs2308161	NED-TGTAGCGGCAATATAGGCAG	GAAAGTTGTGGCTTAACTGG
MID-250	rs16687	NED-ATGGAGCAGTAAAGCAGCAC	GTCACTTTGGTTTTTGCAGG
MID-1802	rs2307998	NED-ACGGTCAACTTTGTAGCTCC	*g*CCAGTTGAGAATCACTGCAC
MID-1607	rs2307803	NED-TGTTGCAGAAGAACTCAACC	GATAAGCACCTAACTCCCAG
MID-1734	rs2307930	PET-TTCGTGTTCTCACACTGTCC	GTGCATCCCATACAACTGAC
MID-406	rs25630	PET-TGGCTGCTGTAGATTGTAGG	*g*ACAAATGGACAACGGCCAAG
MID-1386	rs2307582	PET-AGAGGATCATGGAGACCAAC	*gttt*ATGTTCCAAGTCAGCAGCAC
MID-1726	rs2307922	PET-GGTCCAAATGCACCACAATC	GCTCTGCTATTTTGGTTTGC
MID-3626	rs11267926	PET-TGTTGGTTCTCTCCTTTTCC	GGTGACCCCTTCTTTATCTC
MID-360	rs25584	PET-AGATCAACTGCCAATCTGGG	*g*CTCAAGTGACCAACCCACCT
MID-1603	rs2307799	PET-TTACAATTTCAAGCCTCCGC	GGAGCTGTTAGTCTGAGTAG
MID-2719	rs34541393	PET-GTCAGGAGTCTAGAAACTTC	GGGTGATGAAATGTTCCGAA

* Lowercase italic letters represent nucleotide tails added to the primers.
