# Supplementary material for: Straightforward Inference of Ancestry and Admixture Proportions through Ancestry-Informative Insertion Deletion Multiplexing
Source: PLoS One. 2012 Jan 17;7(1):e29684. doi: 10.1371/journal.pone.0029684 (PMC3260179; doi:10.1371/journal.pone.0029684)
Supplement: Table S2 — Allele frequencies, δ and FST values for the 46 AIM-INDELs in HGDP-CEPH diversity panel population samples from Africa (AFR), Europe (EUR), East Asia (EAS) and Native America (NAM). (PDF) [file pone.0029684.s005.pdf]

Table S2 Allele frequencies, δ and F_{ST} values for the 46 AIM-INDELs in HGDP-CEPH diversity panel population samples from Africa (AFR), Europe (EUR), East Asia (EAS) and Native America (NAM)

MID	rs number	frequency of allele 1				frequency of allele 2				allele frequency differentials (δ)					
		AFR	EUR	EAS	NAM	AFR	EUR	EAS	NAM	AFR/ EUR	AFR/ EAS	AFR/ NAM	EUR/ ASN	EUR/ NAM	EAS/ NAM
MID-1470	rs2307666	0.162	0.563	0.234	0.031	0.838	0.437	0.766	0.969	0.401	0.072	0.131	0.330	0.532	0.202
MID-777	rs1610863	0.390	0.288	0.550	0.844	0.610	0.712	0.450	0.156	0.103	0.160	0.453	0.262	0.556	0.294
MID-196	rs16635	0.538	0.472	0.550	0.063	0.462	0.528	0.450	0.938	0.067	0.012	0.476	0.079	0.409	0.488
MID-881	rs1610965	0.324	0.880	0.932	0.992	0.676	0.120	0.068	0.008	0.556	0.609	0.668	0.053	0.112	0.060
MID-3122	rs35451359	0.552	0.991	1.000	1.000	0.448	0.009	0.000	0.000	0.438	0.448	0.448	0.009	0.009	0.000
MID-548	rs140837	0.338	0.196	0.478	0.172	0.662	0.804	0.522	0.828	0.142	0.140	0.166	0.282	0.024	0.306
MID-659	rs1160893	0.276	0.104	0.463	0.258	0.724	0.896	0.537	0.742	0.172	0.187	0.018	0.358	0.153	0.205
MID-2011	rs2308203	0.162	0.791	0.876	0.906	0.838	0.209	0.124	0.094	0.629	0.714	0.744	0.084	0.115	0.031
MID-2929	rs33974167	0.790	0.655	0.974	0.977	0.210	0.345	0.026	0.023	0.135	0.183	0.186	0.319	0.322	0.003
MID-593	rs1160852	0.695	0.006	0.002	0.008	0.305	0.994	0.998	0.992	0.689	0.693	0.687	0.004	0.001	0.006
MID-798	rs1610884	0.295	0.642	0.157	0.117	0.705	0.358	0.843	0.883	0.347	0.138	0.178	0.485	0.525	0.040
MID-1193	rs2067280	0.178	0.098	0.341	0.141	0.822	0.902	0.659	0.859	0.080	0.163	0.037	0.243	0.043	0.200
MID-1871	rs2308067	0.081	0.326	0.666	0.289	0.919	0.674	0.334	0.711	0.245	0.585	0.208	0.340	0.037	0.377
MID-17	rs4183	0.795	0.338	0.797	0.898	0.205	0.662	0.203	0.102	0.458	0.002	0.103	0.459	0.561	0.102
MID-2538	rs3054057	0.000	0.494	0.024	0.063	1.000	0.506	0.976	0.938	0.494	0.024	0.063	0.470	0.431	0.038
MID-1644	rs2307840	0.229	0.953	0.251	0.195	0.771	0.047	0.749	0.805	0.724	0.023	0.033	0.701	0.757	0.056
MID-3854	rs60612424	0.748	0.013	0.111	0.008	0.252	0.987	0.889	0.992	0.735	0.636	0.740	0.099	0.005	0.104
MID-2275	rs3033053	0.486	0.095	0.775	0.523	0.514	0.905	0.225	0.477	0.391	0.289	0.038	0.680	0.429	0.252
MID-94	rs16384	0.071	0.155	0.059	0.648	0.929	0.845	0.941	0.352	0.084	0.012	0.577	0.096	0.493	0.589
MID-3072	rs34611875	0.100	0.940	0.996	0.977	0.900	0.060	0.004	0.023	0.840	0.896	0.877	0.056	0.037	0.019
MID-772	rs1610859	0.943	0.968	0.465	0.781	0.057	0.032	0.535	0.219	0.025	0.478	0.162	0.503	0.187	0.316
MID-2313	rs3045215	0.110	0.247	0.587	0.730	0.890	0.753	0.413	0.270	0.137	0.478	0.621	0.341	0.483	0.143
MID-397	rs25621	0.771	0.766	0.655	0.344	0.229	0.234	0.345	0.656	0.006	0.116	0.428	0.111	0.422	0.311
MID-1636	rs2307832	0.148	0.785	0.897	0.992	0.852	0.215	0.103	0.008	0.637	0.750	0.845	0.113	0.207	0.095
MID-51	rs16343	0.057	0.642	0.878	0.867	0.943	0.358	0.122	0.133	0.585	0.821	0.810	0.235	0.225	0.011
MID-2431	rs3031979	0.052	0.111	0.373	0.234	0.948	0.889	0.627	0.766	0.058	0.321	0.182	0.263	0.124	0.139
MID-2264*	rs34122827*	0.162	0.465	0.059	0.133	0.838	0.449	0.941	0.867	0.303	0.103	0.029	0.406	0.332	0.074
MID-2256	rs133052	0.000	0.222	0.039	0.836	1.000	0.778	0.961	0.164	0.222	0.039	0.836	0.182	0.614	0.797
MID-128	rs6490	0.024	0.481	0.079	0.047	0.976	0.519	0.921	0.953	0.457	0.055	0.023	0.402	0.434	0.032
MID-15	rs4181	0.195	0.453	0.290	0.836	0.805	0.547	0.710	0.164	0.257	0.095	0.641	0.162	0.383	0.546
MID-2241	rs3030826	0.814	0.293	0.081	0.070	0.186	0.707	0.919	0.930	0.521	0.734	0.744	0.212	0.223	0.010
MID-419	rs140708	0.919	0.810	0.153	0.563	0.081	0.190	0.847	0.438	0.109	0.766	0.357	0.657	0.248	0.410
MID-943	rs1611026	0.176	0.791	0.223	0.398	0.824	0.209	0.777	0.602	0.615	0.047	0.222	0.568	0.393	0.176
MID-159	rs16438	0.752	0.570	0.128	0.734	0.248	0.430	0.872	0.266	0.183	0.624	0.018	0.441	0.165	0.606
MID-2005	rs2308161	0.029	0.671	0.142	0.325	0.971	0.329	0.858	0.675	0.642	0.113	0.297	0.529	0.345	0.183
MID-250	rs16687	0.824	0.715	0.795	0.328	0.176	0.285	0.205	0.672	0.109	0.029	0.496	0.080	0.387	0.467
MID-1802	rs2307998	0.543	0.003	0.007	0.000	0.457	0.997	0.993	1.000	0.540	0.536	0.543	0.003	0.003	0.007
MID-1607	rs2307803	0.424	0.161	0.260	0.555	0.576	0.839	0.740	0.445	0.262	0.164	0.131	0.098	0.393	0.295
MID-1734	rs2307930	0.852	0.835	0.539	0.375	0.148	0.165	0.461	0.625	0.017	0.313	0.477	0.296	0.460	0.164
MID-406	rs25630	0.048	0.823	0.919	0.703	0.952	0.177	0.081	0.297	0.775	0.871	0.656	0.096	0.120	0.216
MID-1386	rs2307582	0.138	0.218	0.336	0.766	0.862	0.782	0.664	0.234	0.080	0.198	0.628	0.118	0.547	0.429
MID-1726	rs2307922	0.176	0.700	0.643	0.758	0.824	0.300	0.357	0.242	0.524	0.466	0.582	0.057	0.058	0.115
MID-3626	rs11267926	0.124	0.699	0.186	0.039	0.876	0.301	0.814	0.961	0.575	0.062	0.085	0.514	0.660	0.147
MID-360*	rs25584*	0.591	0.864	0.116	0.523	0.317	0.136	0.884	0.477	0.273	0.476	0.068	0.748	0.340	0.408
MID-1603	rs2307799	0.081	0.389	0.546	0.820	0.919	0.611	0.454	0.180	0.308	0.465	0.739	0.157	0.431	0.274
MID-2719	rs34541393	0.448	0.345	0.635	0.469	0.552	0.655	0.365	0.531	0.103	0.188	0.021	0.290	0.124	0.167
									mean:	0.349	0.332	0.380	0.282	0.301	0.215

* For these markers an allele 3 was observed with frequency = 1-(allele 1 frequency + allele2 frequency)

(continued on next page)

Table S2 (continued) Allele frequencies, δ and F_{ST} values for the 46 AIM-INDELs in HGDP-CEPH diversity panel population samples from Africa (AFR), Europe (EUR), East Asia (EAS) and Native America (NAM)

MID	rs number	pairwise F_{ST} (single locus and overall at the bottom)						F_{ST} (single locus and mean at the bottom)			
		AFR/ EUR	AFR/ EAS	AFR/ NAM	EUR/ ASN	EUR/ NAM	EAS/ NAM	AFR *vs* Others	EUR *vs* Others	ASN *vs* Others	NAM *vs* Others
MID-1470	rs2307666	0.282	0.012	0.076	0.206	0.432	0.118	0.148	0.301	0.150	0.244
MID-777	rs1610863	0.020	0.046	0.337	0.128	0.454	0.158	0.091	0.170	0.114	0.290
MID-196	rs16635	0.004	-0.003	0.386	0.010	0.297	0.363	0.066	0.062	0.096	0.316
MID-881	rs1610965	0.504	0.622	0.615	0.014	0.070	0.029	0.645	0.300	0.379	0.336
MID-3122	rs35451359	0.477	0.562	0.391	0.009	0.001	0.000	0.665	0.337	0.393	0.299
MID-548	rs140837	0.048	0.035	0.061	0.155	-0.004	0.168	0.044	0.107	0.130	0.103
MID-659	rs1160893	0.094	0.066	-0.005	0.255	0.083	0.077	0.065	0.182	0.175	0.062
MID-2011	rs2308203	0.562	0.683	0.702	0.024	0.039	-0.001	0.650	0.301	0.394	0.322
MID-2929	rs33974167	0.039	0.192	0.129	0.319	0.231	-0.005	0.112	0.266	0.213	0.153
MID-593	rs1160852	0.728	0.778	0.635	0.000	-0.005	0.000	0.845	0.588	0.638	0.534
MID-798	rs1610884	0.211	0.054	0.080	0.404	0.413	0.001	0.138	0.380	0.257	0.215
MID-1193	rs2067280	0.024	0.058	-0.002	0.146	0.004	0.085	0.044	0.098	0.120	0.053
MID-1871	rs2308067	0.153	0.491	0.143	0.205	-0.002	0.241	0.341	0.174	0.333	0.161
MID-17	rs4183	0.342	-0.004	0.031	0.359	0.456	0.029	0.175	0.397	0.230	0.235
MID-2538	rs3054057	0.446	0.014	0.075	0.486	0.319	0.019	0.328	0.557	0.378	0.256
MID-1644	rs2307840	0.727	-0.002	-0.004	0.654	0.786	0.004	0.391	0.643	0.455	0.393
MID-3854	rs60612424	0.765	0.613	0.694	0.068	-0.004	0.058	0.744	0.476	0.456	0.427
MID-2275	rs3033053	0.332	0.171	-0.005	0.625	0.405	0.138	0.215	0.492	0.431	0.201
MID-94	rs16384	0.028	-0.003	0.563	0.048	0.431	0.649	0.198	0.187	0.266	0.610
MID-3072	rs34611875	0.834	0.927	0.855	0.055	0.009	0.016	0.910	0.714	0.775	0.686
MID-772	rs1610859	0.004	0.376	0.111	0.439	0.191	0.173	0.296	0.346	0.430	0.179
MID-2313	rs3045215	0.055	0.365	0.586	0.207	0.379	0.037	0.311	0.217	0.254	0.297
MID-397	rs25621	-0.004	0.028	0.315	0.026	0.312	0.172	0.071	0.078	0.064	0.254
MID-1636	rs2307832	0.571	0.732	0.813	0.046	0.145	0.053	0.704	0.352	0.458	0.432
MID-51	rs16343	0.516	0.787	0.809	0.147	0.109	-0.005	0.675	0.327	0.476	0.367
MID-2431	rs3031979	0.017	0.224	0.138	0.160	0.054	0.037	0.155	0.119	0.182	0.059
MID-2264	rs34122827	0.213	0.058	-0.002	0.407	0.231	0.034	0.143	0.378	0.265	0.143
MID-2256	rs133052	0.188	0.025	0.870	0.149	0.535	0.841	0.419	0.364	0.465	0.778
MID-128	rs6490	0.394	0.022	0.002	0.357	0.327	0.002	0.250	0.445	0.262	0.213
MID-15	rs4181	0.132	0.020	0.576	0.053	0.250	0.436	0.173	0.120	0.152	0.402
MID-2241	rs3030826	0.422	0.731	0.698	0.147	0.126	-0.004	0.639	0.294	0.435	0.346
MID-419	rs140708	0.042	0.723	0.309	0.607	0.141	0.348	0.533	0.482	0.605	0.306
MID-943	rs1611026	0.544	0.003	0.114	0.486	0.288	0.071	0.286	0.471	0.317	0.173
MID-159	rs16438	0.066	0.589	-0.006	0.367	0.050	0.583	0.372	0.262	0.454	0.337
MID-2005	rs2308161	0.588	0.062	0.302	0.462	0.209	0.101	0.361	0.501	0.336	0.199
MID-250	rs16687	0.028	-0.001	0.411	0.015	0.260	0.380	0.096	0.077	0.111	0.359
MID-1802	rs2307998	0.589	0.635	0.486	-0.002	-0.003	-0.001	0.741	0.430	0.476	0.381
MID-1607	rs2307803	0.158	0.057	0.028	0.025	0.312	0.172	0.091	0.109	0.070	0.192
MID-1734	rs2307930	-0.003	0.187	0.402	0.175	0.387	0.048	0.173	0.178	0.168	0.248
MID-406	rs25630	0.739	0.853	0.665	0.040	0.036	0.179	0.757	0.441	0.550	0.357
MID-1386	rs2307582	0.017	0.090	0.579	0.031	0.461	0.298	0.172	0.133	0.128	0.417
MID-1726	rs2307922	0.425	0.347	0.511	0.005	0.002	0.024	0.383	0.153	0.153	0.161
MID-3626	rs11267926	0.495	0.010	0.037	0.430	0.579	0.073	0.275	0.514	0.301	0.326
MID-360	rs25584	0.136	0.471	0.029	0.720	0.267	0.379	0.343	0.533	0.557	0.273
MID-1603	rs2307799	0.213	0.356	0.727	0.045	0.302	0.139	0.352	0.144	0.190	0.342
MID-2719	rs34541393	0.018	0.066	-0.005	0.153	0.027	0.051	0.040	0.100	0.106	0.032
		0.365	0.393	0.443	0.284	0.298	0.220	0.340	0.311	0.312	0.293
